# Supplementary material for: Empirical methods for controlling false positives and estimating confidence in ChIP-Seq peaks
Source: BMC Bioinformatics. 2008 Dec 5;9:523. doi: 10.1186/1471-2105-9-523 (PMC2628906; doi:10.1186/1471-2105-9-523)
Supplement: Additional File 1 — A variety of html documents from the USeq web site detailing the available applications, their best usage, output file type descriptions, command line menus, etc. [file 1471-2105-9-523-S1.zip › USeqUserGuides/JavaDoc.zip!/JavaDoc/bioroot/IdNameMatch.html]

IdNameMatch


|  |  |  |
| --- | --- | --- |
| **Overview** \| **Package** \| **Class** \| **Use** \| **Tree** \| **Deprecated** \| **Index** \| **Help** | |  |
| **PREV CLASS**   **NEXT CLASS** | **FRAMES**    **NO FRAMES**     **All Classes** |  |
| SUMMARY: NESTED \| FIELD \| CONSTR \| METHOD | DETAIL: FIELD \| CONSTR \| METHOD |  |


---


## bioroot Class IdNameMatch

```
java.lang.Object
  bioroot.IdNameMatch
```

---

``` public class IdNameMatch extends java.lang.Object ```

**Author:**
:   nix
    Holder for gene or organism, marker, cell type info

---

**Constructor Summary**

`IdNameMatch(int id,
java.lang.String name)`


| **Method Summary** | |
| --- | --- |
| `static IdNameMatch` | `findMatch(IdNameMatch[] inm, java.lang.String test)` |
| `java.lang.String` | `getCollapsed()` |
| `int` | `getId()` |
| `java.lang.String` | `getName()` |
| `java.lang.String` | `getNameLowerCase()` |

**Methods inherited from class java.lang.Object**

`equals, getClass, hashCode, notify, notifyAll, toString, wait, wait, wait`

**Constructor Detail**

### IdNameMatch

```
public IdNameMatch(int id,
                   java.lang.String name)
```


**Method Detail**

### findMatch

```
public static IdNameMatch findMatch(IdNameMatch[] inm,
                                    java.lang.String test)
```

---


### getId

```
public int getId()
```

---


### getName

```
public java.lang.String getName()
```

---


### getNameLowerCase

```
public java.lang.String getNameLowerCase()
```

---


### getCollapsed

```
public java.lang.String getCollapsed()
```


---


|  |  |  |
| --- | --- | --- |
| **Overview** \| **Package** \| **Class** \| **Use** \| **Tree** \| **Deprecated** \| **Index** \| **Help** | |  |
| **PREV CLASS**   **NEXT CLASS** | **FRAMES**    **NO FRAMES**     **All Classes** |  |
| SUMMARY: NESTED \| FIELD \| CONSTR \| METHOD | DETAIL: FIELD \| CONSTR \| METHOD |  |


---
